# Metformin attenuates plaque-associated tau pathology and reduces amyloid-β burden in APP/PS1 mice

**DOI:** 10.1186/s13195-020-00761-9

**Published:** 2021-02-09

**Authors:** Yanxing Chen, Shuai Zhao, Ziqi Fan, Zheyu Li, Yueli Zhu, Ting Shen, Kaicheng Li, Yaping Yan, Jun Tian, Zhirong Liu, Baorong Zhang

**Affiliations:** 1 Department of Neurology, The Second Affiliated Hospital, School of Medicine, Zhejiang University, Hangzhou, People’s Republic of China; 2 Department of Geriatrics, The First Affiliated Hospital, School of Medicine, Zhejiang University, Hangzhou, People’s Republic of China

**Keywords:** Aβ pathology, Tau pathology, Spread, Metformin, Microglia, Alzheimer’ disease

## Abstract

**Background:**

The neuropathological hallmarks of Alzheimer’s disease (AD) are amyloid-β (Aβ) plaques and neurofibrillary tangles (NFTs). The amyloid cascade theory is the leading hypothesis of AD pathology. Aβ deposition precedes the aggregation of tau pathology and Aβ pathology precipitates tau pathology. Evidence also indicates the reciprocal interactions between amyloid and tau pathology. However, the detailed relationship between amyloid and tau pathology in AD remains elusive. Metformin might have a positive effect on cognitive impairments. However, whether metformin can reduce AD-related pathologies is still unconclusive.

**Methods:**

Brain extracts containing tau aggregates were unilaterally injected into the hippocampus and the overlying cerebral cortex of 9-month-old APPswe/PS1DE9 (APP/PS1) mice and age-matched wild-type (WT) mice. Metformin was administrated in the drinking water for 2 months. Aβ pathology, tau pathology, plaque-associated microgliosis, and autophagy marker were analyzed by immunohistochemical staining and immunofluorescence analysis 2 months after injection of proteopathic tau seeds. The effects of metformin on both pathologies were explored.

**Results:**

We observed tau aggregates in dystrophic neurites surrounding Aβ plaques (NP tau) in the bilateral hippocampi and cortices of tau-injected APP/PS1 mice but not WT mice. Aβ plaques promoted the aggregation of NP tau pathology. Injection of proteopathic tau seeds exacerbated Aβ deposits and decreased the number of microglia around Aβ plaques in the hippocampus and cortex of APP/PS1 mice. Metformin ameliorated the microglial autophagy impairment, increased the number of microglia around Aβ plaques, promoted the phagocytosis of NP tau, and reduced Aβ load and NP tau pathology in APP/PS1 mice.

**Conclusion:**

These findings indicate the existence of the crosstalk between amyloid and NP tau pathology. Metformin promoted the phagocytosis of pathological Aβ and tau proteins by enhancing microglial autophagy capability. It reduced Aβ deposits and limited the spreading of NP tau pathology in APP/PS1 mice, which exerts a beneficial effect on both pathologies.

## Introduction

Alzheimer’s disease (AD) is the most common cause of dementia, posing a heavy burden for society. It is characterized by the deposition of extracellular amyloid plaques and the formation of intracellular neurofibrillary tangles (NFTs). The amyloid cascade theory, which is considered as the dominant hypothesis of AD, states that Aβ accumulation is the initial event in the pathogenesis of AD, resulting in the formation of NFTs and neuronal death of AD [1]. However, accumulating studies suggest a mutual interaction between Aβ and tau pathology. Studies in animal models showed that Aβ plaques could enhance tau seeding and pathology [2–4]. It was found that injection of human AD-brain-derived proteopathic tau (AD-tau) into the brains of plaque bearing transgenic mice facilitated the spreading of tau pathology, which initially appeared as NP tau, and then followed by the formation of NFTs and neuropil threads (NTs) [2]. Another study also observed augmented spreading of tau pathology in 5xFAD mice with injection of AD-tau [3]. *App/MAPT* double-knock-in mice exhibited higher tau phosphorylation than did single *MAPT* knock-in mice, indicating the enhancement of Aβ on tau pathology [4].

It is known that tau pathology correlates more strongly with cognitive decline in AD patients than amyloid pathology [5, 6]. NFTs appear in a hierarchical and stereotypical fashion, beginning in the transentorhinal cortex and later spreading to brain regions such as the hippocampus and, eventually, the cortex [7]. Mounting evidence suggests that tau aggregates recruit monomeric tau into fibrillar aggregates that spread to other brain regions which is called “tau propagation” [8, 9].

In recent years, a link between diabetes and AD has been largely accepted. Type 2 diabetes mellitus (T2DM) is associated with cognitive impairment and an increased risk of AD [10]. Impaired glucose metabolism in the brain is detected early in AD [11], and AD patients have insulin resistance in the brain [12]. Metformin is widely used for the treatment of T2DM. Growing evidence suggests that it might benefit age-related diseases such as cancer, cardiovascular disease, and neurodegenerative diseases, such as AD [13]. However, the effects of metformin on cognition are still controversial. Epidemiological studies have shown that metformin usage is associated with significantly lower risk of cognitive dysfunction in older adults with T2DM in both cross-sectional analysis and in longitudinal analysis [14–16]. An interventional study found that, in overweight patients aged 55 to 90 years with amnestic mild cognitive impairment, those receiving metformin for 12 months had better performance in the selective reminding test than those receiving placebo [17]. However, a case-control study reported that long-term treatment of metformin (≥ 60 prescriptions, an average prescription covers 45–90 days of treatment) was associated with higher risk of AD in T2DM patients over age 65, although there was no consistent trend with increasing number of prescriptions [18]. It was suggested that the effects of metformin on cognition may differ relying on the risk profile of the patients [19]. Mechanistic studies have also been carried out to unveil the effect on metformin on AD pathologies. Protective effect of metformin on cognitive impairment in different models of diabetes has been reported [20–22]. Of note, it is reported that metformin could reduce tau phosphorylation in different mouse models including AD [21, 23, 24]. However, increased insoluble tau species was also reported in another study [25]. Further studies are still in need to elucidate whether metformin could exert beneficial effects on AD pathologies.

In this study, we seeded APP/PS1 mice with tau seeds to investigate the effects of amyloid pathology on tau seeding activity. The effects of metformin on amyloid pathology and tau spreading were also investigated. We observed that Aβ plaque promoted NP tau aggregation. Injection of proteopathic tau seeds exacerbated Aβ deposits and decreased the number of microglia around Aβ plaques. Administration of metformin ameliorated the microglial autophagy impairment, enhanced the activation of microglia around Aβ plaques, promoted the phagocytosis of NP tau, and attenuated Aβ load and NP tau aggregation.

## Methods

### Animals and drug treatment

All procedures were approved by the Institutional Animal Care and Use Committee of Zhejiang University and were performed in accordance with the National Institutes of Health Guide for the Care and Use of Laboratory Animals guidelines for the ethical treatment of animals. Efforts were made to minimize the number of animals used. APP/PS1 transgenic mice were purchased from Model Animal Research Center of Nanjing University (Nanjing, China). Mice were housed in filtered cages in a temperature-controlled room with a 12:12 light/dark cycle with free access to food and water.

At 9 months of age, female APP/PS1 mice were randomly assigned to four groups: (1) APP/PS1 mice injected with brain extract from WT control mice (^WT^BE) and treated with vehicle (ctr+veh group, *n* = 5); (2) APP/PS1 mice injected with brain extract containing tau aggregates from PS19 mice (^PS19^BE) and treated with vehicle (tau+veh group, *n* = 5); (3) APP/PS1 mice injected with ^WT^BE and treated with metformin (ctr+met group, *n* = 5); (4) APP/PS1 mice injected with ^PS19^BE and treated with metformin (tau+met group, *n* = 5). Another group of WT mice injected with ^PS19^BE and treated with vehicle (tau+veh group, *n* = 5) were also included. Mice in the treatment group received 4 mg/ml metformin (Sigma, St. Louis, MO, USA) in the drinking water for 2 months. Mice in the vehicle group received normal drinking water. Drinking bottles were replenished with fresh water or metformin solution every week.

### Preparation of brain extracts

PS19 mice were obtained from the Jackson Laboratory (B6;C3-Tg (Prnp-MAPT*P301S)PS19Vle/J; stock number, 008169, New Harbor, ME, USA). Brain extracts were prepared from end-stage PS19 mice and age-matched WT mice as previously described [26]. Briefly, mice were sacrificed by dislocation of the neck and decapitation. Cerebral cortices and hippocampi were snap frozen in dry ice. Tissues were combined and homogenized at 10% (w/v) in sterile phosphate-buffered saline (PBS), followed by sonication and centrifugation at 3000*g* at 4 °C for 5 min. The supernatant was stored at − 80 °C until use.

### Steoretaxic injection

**Fig. 1:**
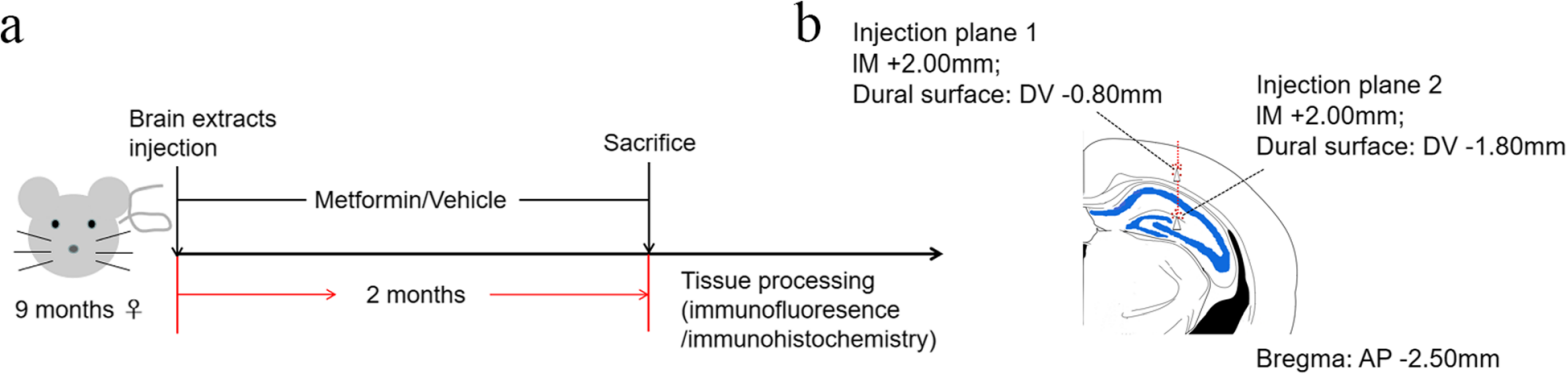
Schematic illustrations of injection sites with coronal planes**. a** Study design. **b** Red dotted line indicates injection path and white arrowheads indicate injection site

Mice were deeply anesthetized with 1.25% Avertin (Sigma, St. Louis, MO, USA). Intracerebral injections were performed as described previously [26] and is shown in Fig. 1. In brief, mice were placed in a stereotaxic frame and a heating blanket with a rectal probe to maintain body temperature at 38 ± 1 °C. Once the skull was exposed and cleaned, a hole was drilled over the appropriate coordinates and brain extracts were unilaterally infused using a Hamilton syringe. The hippocampus (bregma, − 2.50 mm; lateral, + 2.00 mm; and depth, − 1.80 mm) and then the overlying cerebral cortex (bregma, − 2.50 mm; lateral, + 2.00 mm; and depth, − 0.80 mm) were injected with 2.5 μl of inoculum at a flow rate of 1.25 μl/min and the needle was kept in position for additional 3 min before slow withdrawal. Mice were allowed to completely recover on a heating pad before they were returned to their home cages.

### Immunohistochemistry and immunofluorescence

Two months after treatment, mice were deeply anesthetized and transcardially perfused with PBS followed by buffered 4% paraformaldehyde. The whole brains were collected, post-fixed in buffered 4% paraformaldehyde overnight at 4 °C, and dehydrated in buffered 30% sucrose solution. The brains were then cut into 30-μm serial coronal sections on a cryostat and the free-floating sections were preserved in anti-freeze solution (glycerol, ethylene glycol in 0.1 M PBS) at − 20 °C until use.

Immunohistochemistry was performed according to our previously described methods [27]. Briefly, sections were incubated with 0.3% H_2_O_2_ for 20 min and 0.3% Triton X-100 for 15 min at room temperature, washed in PBS, and blocked in 5% normal goat serum with 0.1% Triton X-100 for 30 min. Sections were then incubated overnight with anti-Aβ antibody (6E10) (1:500 dilution; Biolegend, San Diego, CA, USA) at 4 °C. Sections were washed and incubated with horseradish peroxidase-conjugated secondary antibody and visualized with a stable diaminobenzidine/hydrogen peroxide solution. Stained sections were then mounted on microscope slides, dehydrated through graded alcohols, cleared in xylene, and sealed with neutral balsam.

For immunofluorescence, sections were permeabilized with 0.3% Triton X-100, blocked with 5% normal goat serum for 30 min, and then incubated with the following primary antibodies overnight at 4 °C: anti-Aβ antibody (6E10) (1:500 dilution; Biolegend, San Diego, CA, USA), anti-Aβ antibody (4G8) (1:500 dilution; Biolegend, San Diego, CA, USA), anti-AT8 antibody (phospho-tau at Ser202/Thr 205; 1:500 dilution; Thermo Fisher Scientific, MA, USA), anti-AT180 antibody (phospho-tau at Thr231; 1:500 dilution; Thermo Fisher Scientific, MA, USA), anti-phospho-Tau (Ser422) antibody (1:500 dilution; Thermo Fisher Scientific, MA, USA), anti-Iba1 antibody (1:500 dilution; Wako Chemicals, Richmond, VA, USA), and anti-p62 antibody (1:250 dilution; Cell Signaling Technology, Danvers, MA). Normal goat serum in the absence of primary antibody was used as a negative control. The next morning, sections were incubated with an appropriate secondary antibody for 1 h in darkness. Then, the sections were incubated with 4′,6-diamidino-2-phenylindole (DAPI) for 5 min, washed in PBS, and mounted on microscope slides with SlowFade Gold antifade reagent.

Numbers of AT8+ NP tau was analyzed as previously described [2]. In brief, NP tau was defined as a cluster of granule which was AT8+ without a single central nucleus. Four coronal sections of the same reference position from each mouse were selected and measured through blinded manual counting. Quantification of Aβ plaque was performed according to our previously described methods [28]. Briefly, four sections were selected from each mouse using the ImageJ software (NIH, Bethesda, MD, USA). The Aβ plaque burden (% of area) was calculated relative to the total area of the selected region (area% = plaque area/total area selected × 100%). Number of plaque-associated microglia was analyzed as previously described [29]. Briefly, to assign spots to each microglial cell body, the same threshold was applied in all images. 6E10 surfaces were dilated 15 μm, and spots of microglia were counted manually in the expanded area. Any spots completely within or partially in the extended area were counted. The percentage of the number of Iba1+ microglia containing AT8+ aggregates was calculated relative to total number of Iba1+ cells selected. Quantification of p62 was performed as previously described [30]. Briefly, the p62+ area (% of area) was calculated relative to the total area of the selected region (area% = p62+ area/total area selected × 100%). The percentage of p62+/Iba1+ area was calculated relative to the total Iba1+ area selected, and the percentage of the number of Iba1+ microglia containing p62+ aggregates was calculated relative to total number of Iba1+ cells selected. All counts were performed in a blinded fashion.

### Statistical analysis

The GraphPad Prism software (GraphPad Software Inc., San Diego, CA, USA) was used for statistical analysis. For comparisons among various groups, results were analyzed by one-way analyses of variance (ANOVA) followed by the Bonferroni post hoc test. For comparisons between two groups, results were analyzed by the Student’s *t* test. Data are shown as mean ± standard error of the mean (SEM), and *P* values < 0.05 were considered statistically significant.

## Results

### Injection of proteopathic tau seeds induced NP tau aggregation in APP/PS1 mice

**Fig. 2:**
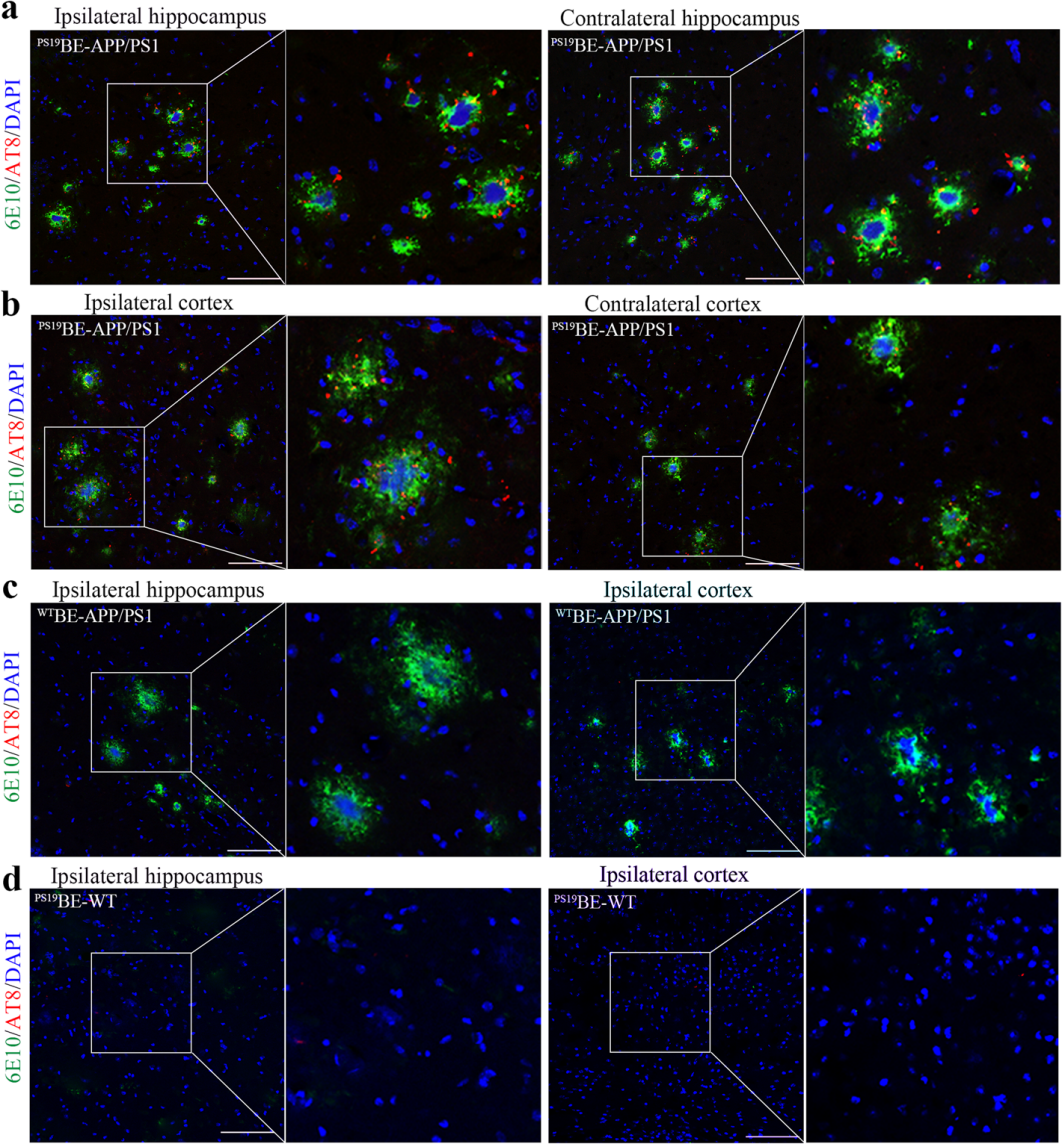
Injection of proteopathic tau seeds induced NP tau aggregation in APP/PS1 mice. **a–d** Representative images of co-staining for 6E10 (green), AT8 (red) in the hippocampus, and cortex of ^PS19^BE-injected APP/PS1 mice (**a**, **b**), ^WT^BE-injected APP/PS1 mice (**c**), and ^PS19^BE-injected WT mice (**d**). Partial magnifications are shown. Scale bar, 100 μm

**Fig. 3:**
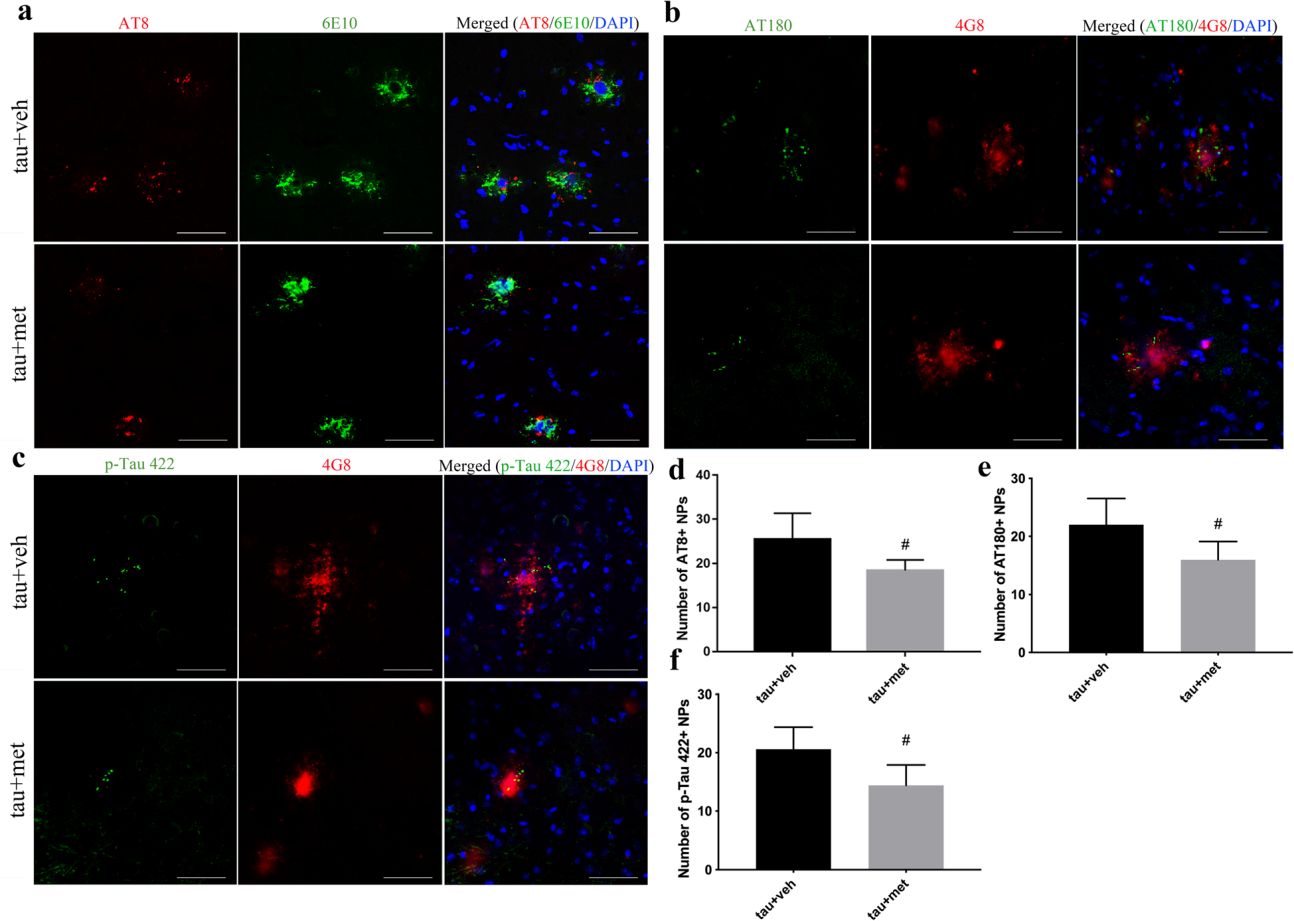
Metformin reduced NP tau aggregation in ^PS19^BE-injected APP/PS1 mice. **a** Representative images of NP tau burden labeled with AT8 (**a**), AT180 (**b**), or p-Tau 422 (**c**) surrounding Aβ burden labeled with 6E10 (**a**) or 4G8 (**b**, **c**) in ^PS19^BE-injected APP/PS1 mice treated with or without metformin. Scale bars, 50 μm. **d–f** Quantification of NP tau in ^PS19^BE-injected APP/PS1 mice treated with or without metformin. Data were analyzed by Student’s *t* test. Values are presented as mean±SEM. *n* = 5 per group. ^#^*P* < 0.05 vs. tau+veh group

Two months following the injection of ^PS19^BE into APP/PS1 mice (Fig. 1), we observed clusters of AT8+ tau particles surrounding 6E10+ Aβ plaques (Fig. 2a, b), resembling NP tau in AD patients. Similar results were observed with antibody 4G8 for Aβ plaques and antibodies AT180 and p-Tau 422 for NP tau staining (Fig. 3). NP tau distributed mainly in the dentate gyrus (DG) and CA3 region of the ipsilateral hippocampus of ^PS19^BE-injected APP/PS1 mice. NP tau was observed not only in the ipsilateral hippocampus and cortex, but also the contralateral hippocampus and cortex in ^PS19^BE-injected APP/PS1 mice, which suggests propagation of tau pathology to the contralateral brain. On the contrary, NP tau pathology was absent in ^WT^BE-injected APP/PS1 mice (Fig. 2c) or ^PS19^BE-injected WT mice (Fig. 2d), indicating that proteopathic tau seeds and Aβ burden are both necessary for the aggregation of NP tau. We did not find NFTs in any group of mice.

### Injection of proteopathic tau seeds exacerbated Aβ burden in APP/PS1 mice

**Fig. 4:**
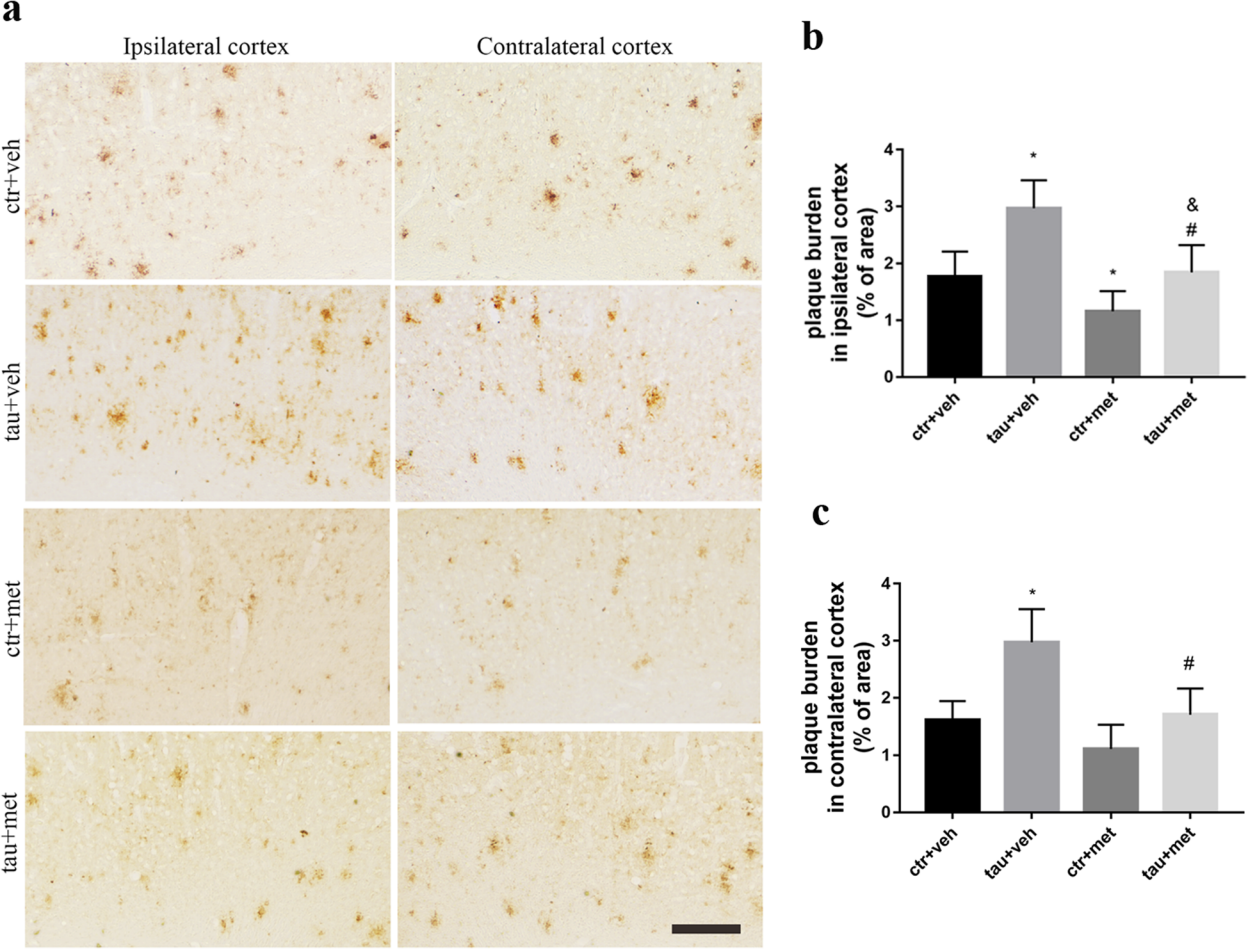
Immunohistochemical analysis of Aβ burden in cortex of APP/PS1 mice**. a** Representative images of 6E10-labeled Aβ burden in the ipsilateral and contralateral cortices of APP/PS1 mice. Scale bars, 200 μm. **b, c** Quantification of percent area covered by 6E10 staining in the ipsilateral cortex (**b**) and contralateral cortex (**c**) of the APP/PS1 mice. Data were analyzed by one-way ANOVA followed by the Bonferroni post hoc test. Values are presented as mean±SEM. *n* = 5 per group. ^*^P < 0.05 vs. ctr+veh group; ^#^*P* < 0.05 vs. tau+veh group; ^&^*P* < 0.05 vs. ctr+met group

**Fig. 5:**
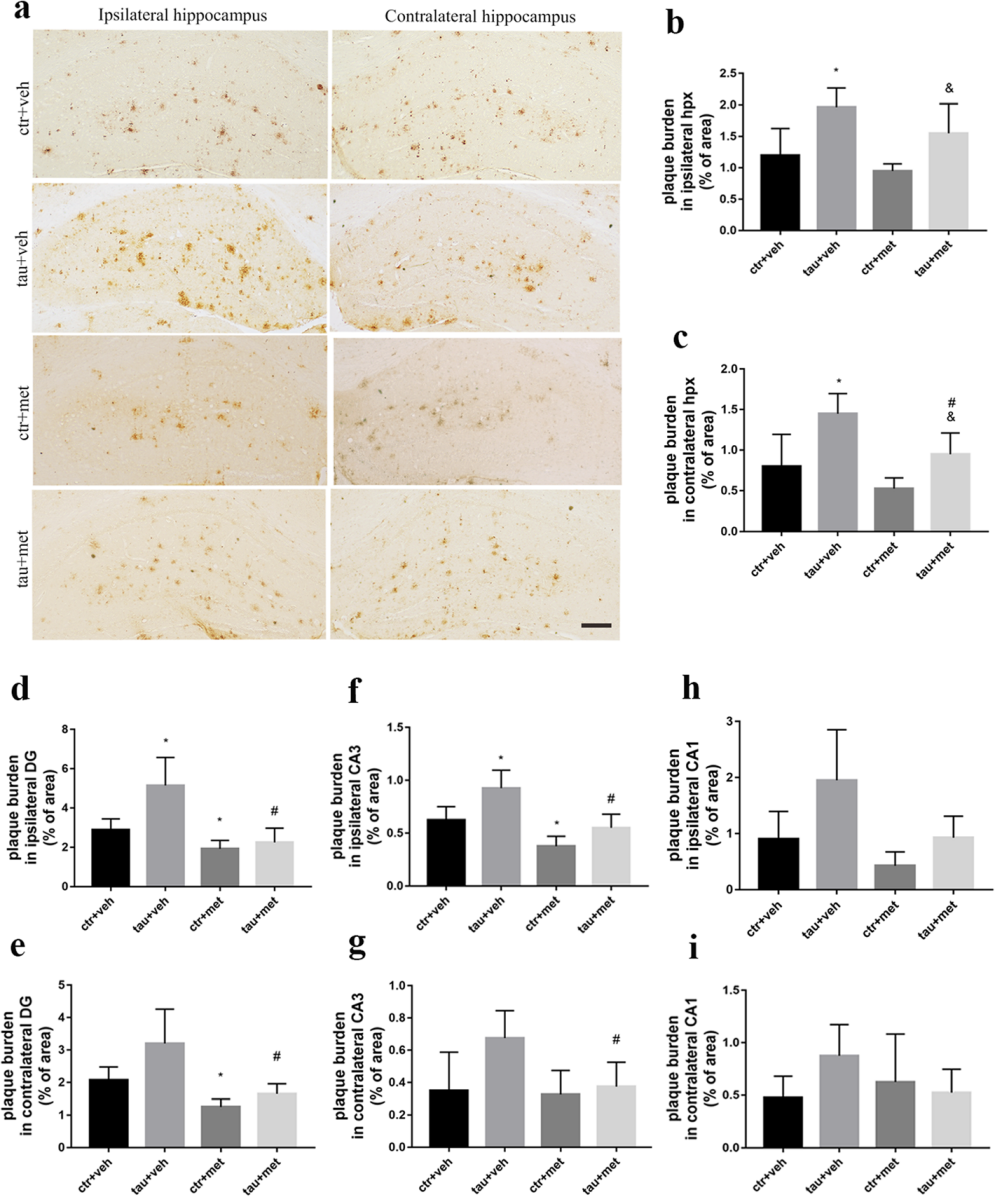
Immunohistochemical analysis of Aβ burden in hippocampus of APP/PS1 mice. **a** Representative images of 6E10-labeled Aβ burden in the ipsilateral and contralateral hippocampus of APP/PS1 mice. Scale bars, 200 μm. **b**, **c** Quantification of percent area covered by 6E10 staining in the ipsilateral hippocampus (hpx) (**b**) and contralateral hippocampus (**c**) of the APP/PS1 mice. **d–i** Quantification of percent area covered by 6E10 staining in the ipsilateral DG (**d**), CA3 (**f**), CA1 (**h**), contralateral DG (**e**), CA3 (**g**), and CA1 (**i**) of the hippocampi of APP/PS1 mice. Data were analyzed by one-way ANOVA followed by the Bonferroni post hoc test. Values are presented as mean±SEM. *n* = 5 per group. ^*^P < 0.05 vs. ctr+veh group; ^#^*P* < 0.05 vs. tau+veh group; ^&^*P* < 0.05 vs. ctr+met group

To better understand the relationship between Aβ pathology and tau pathology, we examined the Aβ plaque burden in APP/PS1 mice. We found much more Aβ burden in the bilateral cortices (Fig. 4a) and hippocampi (Fig. 5a) of ^PS19^BE-injected APP/PS1 mice than that of ^WT^BE-injected APP/PS1 mice. Moreover, increased Aβ plaque burden was especially obvious in the dentate gyrus and CA3 subfields of the ipsilateral hippocampus of ^PS19^BE-injected APP/PS1 mice (Fig. 5d–h).

### Injection of proteopathic tau seeds reduced microgliosis around Aβ plaques in APP/PS1 mice

**Fig. 6:**
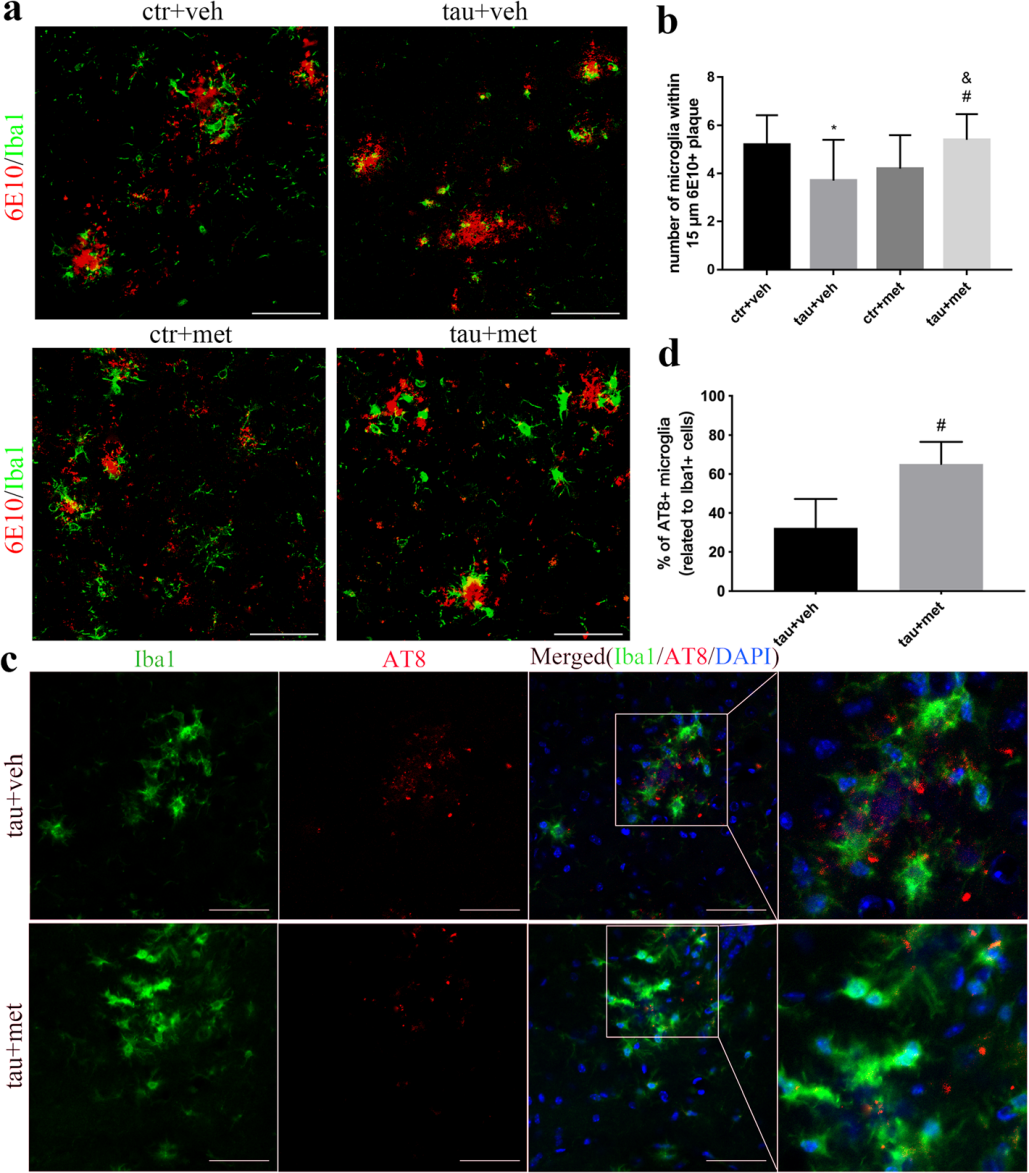
Immunofluorescence analysis of microglia in relation with Aβ plaques and NP tau pathologies in APP/PS1 mice. **a** Representative confocal images of Iba1+ microglia (green) surrounding 6E10+ Aβ plaques (red) in the brains of APP/PS1 mice. Scale bars, 100 μm. **b** Quantification of the number of microglial cells surrounding plaques in APP/PS1 mice. Data were analyzed by one-way ANOVA followed by the Bonferroni post hoc test. Values are presented as mean±SEM. *n* = 5 per group. ^*^P < 0.05 vs. ctr+veh group; ^#^*P* < 0.05 vs. tau+veh group; ^&^*P* < 0.05 vs. ctr+met group. **c** Representative confocal images of Iba1+ microglia (green) and AT8+ NP tau (red) in the brains of APP/PS1 mice. Scale bars, 50 μm. **d** Percentage of AT8+ microglial cells with respect to total Iba1+ microglial cells in the in ^PS19^BE-injected APP/PS1 mice treated with or without metformin. Data were analyzed by Student’s *t* test. Values are presented as mean±SEM. *n* = 5 per group. ^#^*P* < 0.05 vs. tau+veh group

To explore the underlying mechanisms of increased Aβ accumulation, we investigated the involvement of microglia in APP/PS1 mice. We observed significantly decreased numbers of microglia surrounding Aβ plaques in the ^PS19^BE-injected APP/PS1 mice as compared to the ^WT^BE-injected APP/PS1 mice (Fig. 6a, b). The results showed that injection of proteopathic tau seeds decreased plaque-associated microgliosis.

### Metformin reduced Aβ burden in APP/PS1 mice

To examine the effect of metformin on Aβ plaques in the brain, we analyzed Aβ plaque deposits in the brains of APP/PS1 mice. In the ^PS19^BE-injected APP/PS1 mice, metformin significantly reduced Aβ plaques in bilateral cortices and the contralateral hippocampus of the mice (Figs 4 and 5). Aβ plaque burden was further analyzed in the subfields of the hippocampus. We found decreased Aβ burden in bilateral dentate gyrus and CA3 subfields of the hippocampus of metformin-treated mice than that of the vehicle-treated mice. No significant differences of Aβ burden in the CA1 subfield of the hippocampus between the groups were observed. In the ^WT^BE-injected APP/PS1 mice, metformin decreased Aβ burden in the bilateral dentate gyrus and ipsilateral CA3 subfields of hippocampus (Fig. 5). These results demonstrated that metformin treatment ameliorated Aβ plaque burden as well as pathologic tau-induced exacerbation of Aβ accumulation in APP/PS1 mice.

### Metformin limited the spreading of NP tau aggregation in ^PS19^BE-injected APP/PS1 mice

To explore the effect of metformin on the spreading of NP tau aggregation in ^PS19^BE-injected APP/PS1 mice, the amounts of NP tau labeled with antibodies AT8, AT180, or p-Tau 422 surrounding individual Aβ plaques labeled with antibodies 6E10 and 4G8 were quantified. The numbers of NP tau in metformin-treated mice were reduced in comparison to the vehicle-treated mice (Fig. 3), which implied that metformin limited the spreading of tau pathology in APP/PS1 mice.

### Metformin enhanced plaque-associated microgliosis in APP/PS1 mice

To explore the possible mechanisms underlying the effects of metformin on Aβ pathology, we investigated the involvement of microglia. Administration of metformin enhanced the plaque-associated microgliosis (Fig. 6a, b). Moreover, we found more activated microglial cells with larger, rounder somata and short, thick extensions surrounding Aβ plaques in the brains of the metformin-treated mice. Altogether, the data suggests that metformin effectively promoted the activation of microglia in ^PS19^BE-injected APP/PS1 mice.

### Metformin promoted the phagocytosis of NP tau aggregates by microglia in ^PS19^BE-injected APP/PS1 mice

To explore the mechanisms underlying the decreased NP tau pathology in metformin-treated mice, co-localization between Iba1+ microglia and AT8+ seeds were investigated. We found internalized NP tau in microglia (Fig. 6c, d), indicating that microglia took up tau seeds in ^PS19^BE-injected APP/PS1 mice. This observation implies that metformin may promote the phagocytosis of tau seeds by microglia, thus reducing the NP tau pathology.

### Metformin reduced accumulation of autophagy-related protein in APP/PS1 mice

**Fig. 7:**
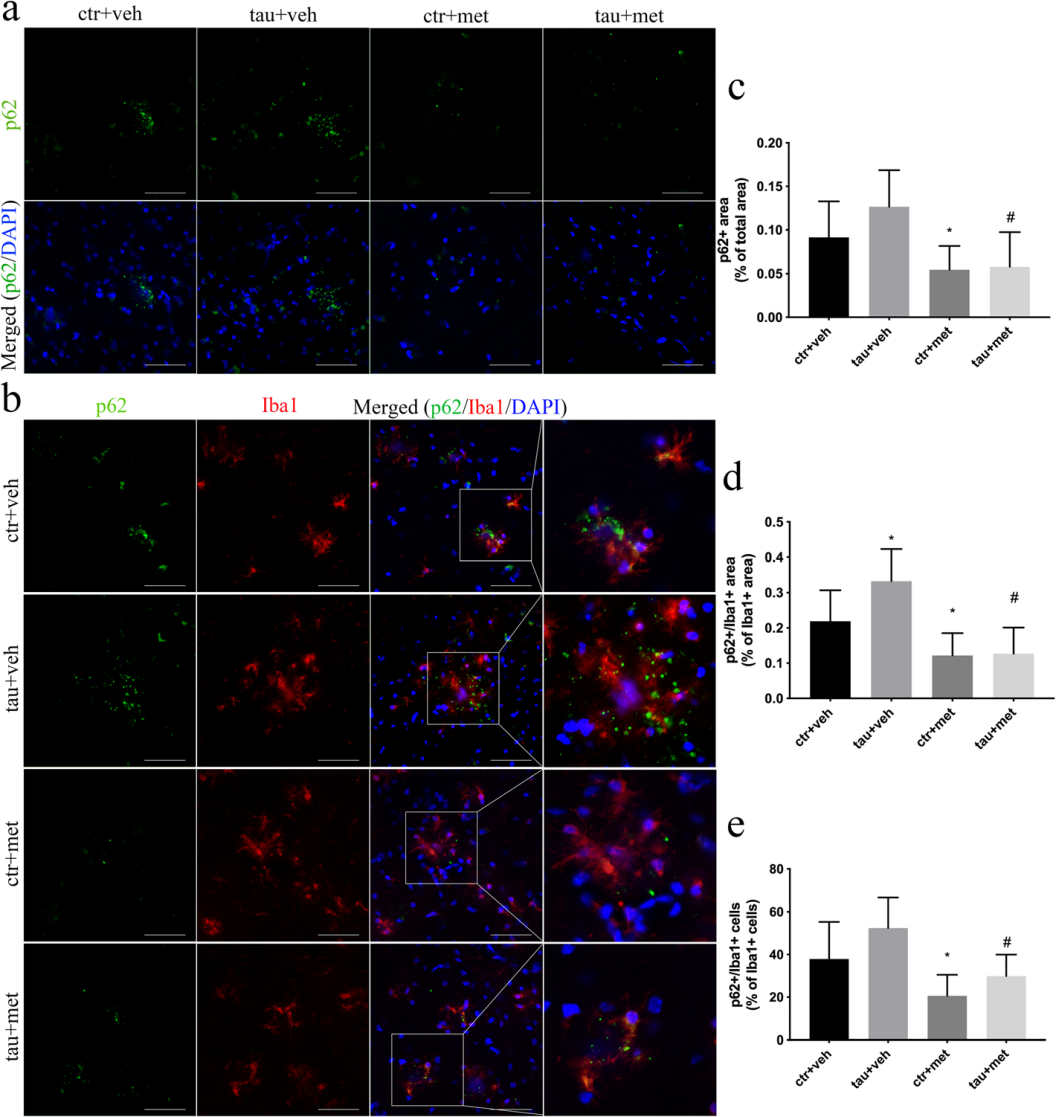
Metformin reduced accumulation of autophagy-related protein in microglia in APP/PS1 mice. **a** Representative images of p62+ aggregates (green) in the brains of APP/PS1mice. Scale bars, 50 μm. **b** Confocal images of Iba1+ microglia (red) and p62+ aggregates (green) in the brains of APP/PS1 mice. Scale bars, 50 μm. **c** Quantification of percent area covered by p62 staining in APP/PS1 mice. Data were analyzed by one-way ANOVA followed by the Bonferroni post hoc test. Values are presented as mean±SEM. *n* = 5 per group. **d**, **e** Quantitative analysis of p62+ aggregates in microglia, expressed as the percentage of Iba1+ area that is also p62+ (**d**) and the percentage of the number of Iba1+ microglia containing p62+ aggregates (**e**). Data were analyzed by one-way ANOVA followed by the Bonferroni post hoc test. Values are presented as mean±SEM. *n* = 5 per group. ^*^P < 0.05 vs. ctr+veh group; ^#^*P* < 0.05 vs. tau+veh group; ^&^*P* < 0.05 vs. ctr+met group

Autophagy dysregulation has been reported in AD brain, which contributes to the formation of AD pathologies [31]. Therefore, we investigated the involvement of autophagy in the accumulation of Aβ plaques and NP tau pathology in APP/PS1 mice. Protein p62 is an autophagy receptor specifically degraded by autophagy, which is an important autophagy marker [32]. Accumulation of p62+ *puncta* was easily observed in vehicle-treated APP/PS1 mice (Fig. 7a, c), which co-localized with Iba1+ microglia (Fig. 7b). Metformin significantly reduced the accumulation of p62 in both ^WT^BE-injected and ^PS19^BE-injected APP/PS1 mice (Fig. 7a, c). Besides, p62 immunoreactivity on Iba1+ microglia was also significantly decreased in metformin-treated APP/PS1 mice (Fig. 7b, d, e).

## Discussion

In the current study, we explored the relationship between Aβ accumulation and the propagation of tau pathology and assessed the effects of metformin on both pathologies in APP/PS1 mice. We observed seeded NP tau aggregates in ^PS19^BE-injected APP/PS1 mice but not in ^PS19^BE-injected WT mice or ^WT^BE-injected APP/PS1 mice. Increased Aβ plaque burden in the cortex and hippocampus and reduced number of microglia around Aβ plaques were observed in ^PS19^BE-injected APP/PS1 mice. Administration of metformin reduced the accumulation of p62 in microglia, increased the number of microglia around Aβ plaques, promoted the internalization of NP tau by microglia, and reduced Aβ pathology as well as NP tau pathology.

The amyloid cascade theory of AD etiology states that the accumulation of Aβ plaque precipitates tau pathology [1]. In the amyloid cascade hypothesis, Aβ is described as the upstream of tau aggregation, and tau is considered to be the “bullet” of Aβ [33], responsible for the cytotoxic, dystrophic, and functional effects of Aβ [33–36]. This hypothesis is strongly supported by many studies, which have shown that Aβ pathology could facilitate the progression of tau pathology [37–40]. In the current study, we found that injection of brain extract containing tau aggregates into the brains of APP/PS1 mice induced the formation of NP tau aggregation surrounding Aβ plaques. However, no NP tau pathology was observed in the brains of WT mice after injection of proteopathic tau seeds, suggesting that the presence of Aβ pathology was necessary for the aggregation of NP tau pathology. A previous study also demonstrated enhancement of tau spreading in the cortex of 5xFAD mice after intracerebral injection of tau seeds [3]. A recent study showed that NP tau pathology was detected 3 months after injection of tau seeds into 8-month-old 5xFAD mice and NFT pathology was found 6 months after injection [2]. It is proposed that, at the early seeding stage, Aβ plaques induce the accumulation of endogenous tau within dystrophic neurites surrounding the plaques, leading to the rapid recruitment of proteopathic tau seed into aggregates as NP tau. At this stage, insufficient amounts of proteopathic tau seeds are available to translocate into the neuronal soma dendrites to form NFTs [2]. Consistently, NP tau but not NFT pathology was observed 2 months after injection of ^PS19^BE into the APP/PS1 mice, which is in line with this hypothesis.

In our experiment, proteopathic tau seeds were injected into the dentate gyrus and the overlying cortex of the APP/PS1 mice. Two months after injection, NP tau aggregates were mainly distributed in the dentate gyrus and CA3 region of the ipsilateral hippocampus and overlying cortex of the ^PS19^BE-injected APP/PS1 mice. Small amounts of NP tau aggregates were also detected in the contralateral hippocampus and cortex, indicating that tau pathology had progressed into the contralateral hemisphere. Many studies have demonstrated the trans-synaptic propogation of tau pathology [26, 41, 42]. The hippocampal trisynaptic circuitry (perforant pathway-DG-CA3-CA1) has been well clarified by many studies [43]. In the hippocampal synaptic network, CA3 neurons receive inputs from the DG via mossy fiber (MF) and send axons to the CA1 neurons via Schaffer Collateral Pathway (SC) [43]. Thus, it is possible that NP tau pathology was formed in the DG area and then spread to the CA3 subfield through the synaptic connectivity and later propagated into the contralateral hemisphere.

In the current study, Aβ pathology was not seen in the ^PS19^BE-injected WT mice. ^WT^BE-injected APP/PS1 mice did not show any increase in Aβ pathology. These observations suggest that both APP and tau are necessary for the increase in tau seed-related Aβ aggregation. Interestingly, in ^PS19^BE-injected APP/PS1 mice, increased Aβ plaque burden was observed in bilateral cortices and hippocampi, which coincide with the distribution of NP tau aggregates. This observation implies that NP tau pathology can lead to the deterioration of Aβ pathology, challenging the amyloid cascade hypothesis which posits that Aβ acts exclusively upstream of tau pathology and tau pathology is simply a downstream effect of amyloid production in AD. However, the underlying mechanism involved in the accelerated Aβ deposition in the ^PS19^BE-injected APP/PS1 mice remains unclear. Only a few studies have focused on the effects of tau pathology on Aβ accumulation. A previous study showed a fivefold increase of Aβ plaque burden in the brains of double transgenic mice expressing mutated APP and tau in comparison with the mice expressing mutated APP only [44]. Another study demonstrated that tau deletion led to a reduction of Aβ plaque by 50% in mice expressing mutated APP and PS1 [45]. These data indicate that Aβ and tau may have a positive feedback loop, highlighting synergistic interactions in the development of both lesions. But it is still unknown whether the tau pathology or other related pathological changes accompanied the development of NP, like neuroinflammation, leads to the increase accumulation of Aβ plaque. It has been shown that tau pathology could induce neuroinflammation, leading to the upregulation of pro-inflammatory cytokines (IL-6, IL-1β, TNFα) through NF-κB and MAPK signaling pathways [46]. Therefore, further studies are needed to confirm the extent of neuroinflammation induced by NP tau development and its involvement in the accumulation of Aβ pathology.

Given that the effects of metformin on cognition and AD-related pathologies remain to be determined, we investigated whether metformin could prevent the propagation of Aβ and tau pathology in ^PS19^BE-injected APP/PS1 mice. We found decreased Aβ burden as well as reduced NP tau pathology in the brains of ^PS19^BE-injected APP/PS1 mice treated with metformin, implying that metformin could inhibit the progression of both pathologies. Consistently, it was found that metformin decreased both Aβ deposits and soluble Aβ in APP/PS1 mice [47].

As the immune resident cells of the brain, microglia constantly scan their environment for pathogens [48, 49]. Once activated, microglia can phagocytize cellular debris, produce growth factors, and promote nerve repair. On the other hand, excessive activation of microglia may lead to inflammatory injury to the brain [50]. At early stages of AD, microglia can accumulate around Aβ plaques and phagocytize Aβ plaques [51], as well as tau [52]. However, if microglia failed to neutralize the seeding activity of tau seeds efficiently, the proteopathic tau seeds would be released from microglia, leading to the acceleration of tau transmission [52]. The diminished phagocytosis of microglia could also lead to the accumulation of both pathologies. Here, we observed decreased plaque-associated microgliosis in ^PS19^BE-injected APP/PS1 mice, which might result from the toxicity induced by seeded tau pathology. Administration of metformin increased the number of microglia around Aβ plaques, enhancing plaque-associated microgliosis. We found internalized NP tau inside microglia in the metformin-treated ^PS19^BE-injected APP/PS1 mice, which implies that metformin may promote the phagocytosis of tau seeds by microglia, thus reducing the NP tau pathology. These results indicate that metformin may reduce both pathologies by enhancing the phagocytosis of microglia. Consistently, studies have shown that microglia are capable of taking up tau [52, 53]. It was shown that TREM2-facilitated plaque-associated microgliosis prevented tau transmission [29]. Thus, it is conceivable that metformin might promote the microglial phagocytosis of Aβ plaque and tau aggregates, limiting the propagation of Aβ and tau pathology. On the other hand, microglia constantly switch between pro-inflammatory and anti-inflammatory phenotypes under pathological stimulation [54]. Metformin has been shown to decrease the production of pro-inflammatory cytokines in different AD animal models [47, 55]. Thus, it would be of great interest to further investigate the involvement of neuroinflammatory responses in the reduction of Aβ and tau pathologies with metformin treatment.

Autophagy is a catabolic mechanism of bulk degradation. Pathological proteins and dysfunctional organelles are engulfed to form the autophagosome, which subsequently fuses with lysosomes to form autolysosomes, leading to degradation of autophagic cargoes [56]. Autophagy dysfunction has been observed in AD brain, contributing to the accumulation of pathological protein aggregates [57]. Metformin is an activator of adenosine monophosphate (AMP)-activated protein kinase (AMPK) [58, 59], the activation of which could inhibit the activity of mechanistic target of rapamycin complex 1 (mTORC1) and activate the unc-51 like autophagy activating kinase 1 (ULK-1) [57], hence activating the downstream autophagy pathway. Accumulation of the autophagic substrate p62 in microglia indicates the dysfunction of autophagy degradation [30]. In the current study, we observed accumulation of p62 in microglial cells in the brains of APP/PS1 mice, suggesting the impairment of autophagy. Administration of metformin significantly reduced the accumulation of p62 in microglia, indicating that metformin may ameliorate the microglial autophagy impairment. Consistently, metformin has been shown to increase the microglial capability of phagocytosis in AMPK-dependent manner in rat primary microglia [60]. Pharmacologically inhibiting mTORC1 signaling with rapamycin increased autophagy and ameliorated Aβ and tau pathology [61]. Therefore, it is conceivable that metformin might promote the engulfment of Aβ and tau pathology by enhancing the microglial autophagy capability.

Our study showed that metformin limited the propagation of Aβ and NP tau pathology in APP/PS1 mice. However, opposing studies have also been reported. It was found that metformin increased the generation of Aβ in various cell models [62, 63]. These results were observed in cell lines overexpressing APP which is quite different from in vivo condition. In our study, although we could not rule out the effects of metformin on Aβ production in neurons, the reduced Aβ deposition we observed could be resulted from increased microgliosis and the promoted autophagic flux in microglia. Another study also showed increased protein phosphatase 2A (PP2A) level and thus decreased AT8+ staining in P301S mice treated with metformin [25], which is similar to what was found in this study. However, increased insoluble tau species were observed in metformin-treated P301S mouse brain. And the underlying mechanism was unknown. In the current study, we showed decreased NP tau in tau-seeded APP/PS1 mice, suggesting that metformin could interfere with the propagation of tau pathology. These results indicate the potential time-window for metformin treatment as a therapeutic drug for AD. Metformin might benefit mild cognitive impairment (MCI) or early-stage AD patients. However, further clinical trials are undoubtedly needed to confirm this assumption.

Limitations of the current study include the lack of the behavioral studies and biochemical experiments following injection of proteopathic tau seeds into the APP/PS1 mice. Given the main aim of the present study is to explore the relationship between amyloid and tau pathology in AD and to determine the effects of metformin on amyloid and tau pathologies, we did not perform behavioral tests or biochemical experiments of these mice. Further studies including behavioral tests are needed to verify the effects of metformin on the cognitive deficits of tau-seeded APP/PS1 mice. Biochemical studies, investigating the activation of AMPK signaling pathway, the involvement of neuroinflammation and autophagic flux in both microglia and neurons, and the phosphorylation of tau at different epitopes and the activation of related tau phosphatase and kinases, will help demonstrate the underlying mechanisms of the beneficial effects of metformin on amyloid and tau pathologies.

## Conclusion

In conclusion, the present study provides novel evidence that proteopathic tau seeds exacerbated Aβ pathology in APP/PS1 mice and Aβ plaques promoted the aggregation of NP tau pathology. Metformin ameliorated microglial autophagy impairment, promoted the phagocytosis of pathological Aβ and tau proteins, and reduced both Aβ deposits and NP tau pathology in tau-seeded APP/PS1 mice. Our findings highlight a synergistic interaction between Aβ and tau pathology and demonstrate that metformincould limit the propagation of Aβand NP tau pathology by enhancing microglial autophagy activity.

## Data Availability

The datasets used and/or analyzed during the current study are available from the corresponding authors on reasonable request.

## Abbreviations

AD: Alzheimer’s disease
Aβ: Amyloid-β
NFTs: Neurofibrillary tangles
APP/PS1: APPswe/PS1DE9
WT: Wild-type
NP tau: tau aggregates in dystrophic neurites surrounding Aβ plaques
AD-tau: AD-brain-derived proteopathic tau
NTs: Neuropil threads
T2DM: Type 2 diabetes mellitus
^WT^BE: Brain extract from WT control mice
^PS19^BE: Brain extract containing tau aggregates from PS19 mice
PBS: Phosphate-buffered saline
DAPI: 4′,6-Diamidino-2-phenylindole
ANOVA: One-way analyses of variance
SEM: Standard error of the mean
DG: Dentate gyrus
MF: Mossy fiber
SC: Schaffer Collateral Pathway
AMPK: Adenosine monophosphate (AMP)-activated protein kinase
PP2A: Protein phosphatase 2A
mTORC1: Mechanistic target of rapamycin complex 1
ULK-1: Autophagy activating kinase 1
MCI: Mild cognitive impairment
